# 2633. Nasal Colonization Status of Respiratory Viruses in Patients During the COVID-19 Epidemic Period at a Tertiary Hospital in Japan

**DOI:** 10.1093/ofid/ofad500.2245

**Published:** 2023-11-27

**Authors:** Tomohiro Katsuta, Ryo Niiya, Yukitsugu Nakamura, Naoki Shimizu

**Affiliations:** St. Marianna University School of Medicine, Kawasaki, Kanagawa, Japan; St. Marianna University School of Medicine, Kawasaki, Kanagawa, Japan; St Marianna University School of Medicine, Kawasaki, Kanagawa, Japan; St. Marianna University School of Medicine, Kawasaki, Kanagawa, Japan

## Abstract

**Background:**

Few reports have comprehensively investigated the frequency of respiratory virus nasal colonization in both children and adults. Since the coronavirus disease 2019 (COVID-19) pandemic in 2020, St. Marianna University Hospital has used the multiplex polymerase chain reaction (PCR) test (FirmArray^®^ RP2.1 Panel) to diagnose symptomatic patients and admission screening of asymptomatic patients. Thus, we have obtained comprehensive multiplex PCR test results and analyzed the nasal colonization status of respiratory viruses in patients during the COVID-19 epidemic period.

**Methods:**

We conducted a retrospective analysis of the nasal colonization status of respiratory viruses in patients during the COVID-19 epidemic period at St. Marianna University Hospital in Japan, a tertiary hospital with 1,175 beds, including 90 beds for pediatric patients. The results of multiplex PCR tests from August 2020 to January 2023 were analyzed.

**Results:**

A total of 15,107 patients were included in this survey. The patient age groups were < 20 years (1,788, 11.8%), 20–59 years (4,399, 29.1%), and ≥60 years (8,920, 59.1%). The positivity rates for severe acute respiratory syndrome coronavirus 2 were 14.8%, 10.2%, and 6.0%, respectively; rhinovirus or enterovirus 26.2%, 1.9%, and 0.7%, respectively; respiratory syncytial virus 7.1%, 0.4%, and 0.3%, respectively; parainfluenza virus type 3 3.6%, 0.3%, and 0.2%, respectively; and adenovirus 3.1%, 0.3%, and 0.1%, respectively, were detected significantly in children, and to some extent in adults.

**Conclusion:**

Despite various restrictions during the COVID-19 pandemic period, children as well as adults had a certain degree of respiratory virus colonization in the nasal cavity. This result suggests that asymptomatic or mildly symptomatic adults may be spreaders of some viral infections to children.

**Disclosures:**

**All Authors**: No reported disclosures

